# Oleogels—Their Applicability and Methods of Characterization

**DOI:** 10.3390/molecules26061673

**Published:** 2021-03-17

**Authors:** Eckhard Flöter, Till Wettlaufer, Valentina Conty, Maria Scharfe

**Affiliations:** Department of Food Process Engineering, TU Berlin, 13353 Berlin, Germany; till.wettlaufer@tu-berlin.de (T.W.); v.conty@tu-berlin.de (V.C.); maria.scharfe@tu-berlin.de (M.S.)

**Keywords:** non-triacylglyceride structuring, oil binding capacity, incremental structure contribution, oleogel

## Abstract

Oleogels or, more precisely, non-triglyceride structured lipid phases have been researched excessively in the last decade. Yet, no comprehensive knowledge base has emerged, allowing technology elevation from the laboratory bench into the industrial food application. That is partly due to insufficient characterization of the structuring systems studied. Examining a single composition decided upon by arbitrary methods does not stimulate progress in the research and technology area. A framework that gives much better guidance to product applications can easily be derived. For example, the incremental structure contribution concept is advocated as a parameter to compare the potency of structuring systems. These can straightforwardly be determined by combining solubility data and structural measurements in the recommended manner. The current method to determine the oil-binding capacity suffers from reproducibility and relevance. A newly developed method is suggested to overcome these shortcomings. The recommended new characterization of oleogels should contribute to a more comprehensive knowledge base necessary for product innovations.

## 1. Introduction

In our diet and the typical habits to prepare food, vegetable fats and oils play a major role. The properties of different fat phases cover a wide range, satisfying the necessities of many applications. In a first cut, one could distinguish between the applications of either liquid or structured lipid phases. It is fair to consider the first as a straightforward application of seed oils. The criteria to choose raw materials for a specific application are more than just economical. Obviously, sourcing in terms of price, availability, implying here volume and flexibility of origin, is essential. The fatty acid composition of the fat phase relates to chemical stability and nutritional quality. Lastly, somewhat less defined characteristics such as sustainability, carbon footprint, and general image play an increasingly important role. However, liquid lipid phases shall not be the emphasis of this contribution. The same criteria also apply to the selection of the raw materials to compose structured lipid phases. Structured lipid phases are related to either fat originating from a single source which by themselves already have a semi-solid character at ambient temperature, e.g., palm oil, coconut oil, or cocoa butter, or are composed of oils that are subject to structuring or gelation through a structuring agent. Regarding the food market’s current situation, structured lipid phases are almost exclusively based on fat crystal networks. To supply a three-dimensional scaffolding that can bind liquid seed oils and generate macroscopic hardness, the fats preferentially have a limited solubility at ambient and/or storage temperatures. These high-melting fats, also referred to as hard-stocks, are inevitably related to high levels of saturated fatty acids. The discussion on fatty acid composition is deceptive concerning the physical properties of fats and oils. Properties such as melting point, solubility, and complex crystallization behavior are driven by the triacylglycerols (TAGs), which are triplets of fatty acid moieties esterified to a glycerol backbone [1]. The design of the functionality of structuring high melting fats can be achieved by generating the desired TAG mixture through the application of oil modification techniques. Details of the different routes, hydrogenation, interesterification, fractionation, and direct mixing of hard-stocks can be found elsewhere [2,3]. In summary, the in-depth understanding of the fat composition’s interplay and process conditions to generate desired final or intermediate product properties was accumulated over many decades. For further details on fat crystal networks, one can be referred to other contributions within this issue or other works [4].

Essentially, the triglyceride-based structuring is based on the arrangement of the primary fat crystals into a space-filling three-dimensional lattice. These lattices are capable of immobilizing a liquid oil phase. The split between solid and liquid phases can vary greatly depending on the application at hand. It should be pointed out that the nature of primary crystals, their mutual interaction, and the structure build-up from crystal agglomerates can considerably be influenced by the selection of the molecular TAG composition and processing conditions and the presence of other materials [3,5,6,7,8,9]. Obviously, the variation of these parameters allows altering the potential of the three-dimensional network to immobilize oil. Furthermore, fat crystal networks are prone to change over time. Processes such as Ostwald ripening, relaxation of the network, or simple re-crystallization can result in significant macroscopic changes such as changes in the macroscopic hardness or the development of phenomena as chocolate bloom [1,9]. The particle gel that triglyceride crystals supply delivers functionalities in food products beyond the immobilization of liquid oils. These are stabilizing interfaces, bubbles, or droplets, providing macroscopic hardness, and a specific product disintegration behavior, which possibly accounts for cooling sensation through melting, flavor release characteristics, and rheological sensations such as lubrication. 

Unfortunately, fat crystals are intrinsically related to increased levels of saturated fatty acids (SaFA) in a lipid phase. The increased consumption of these fatty acids (SaFA) has been discouraged for reasons of cardiovascular health. How far the health benefit assigned to the substitution of saturated fatty acids by polyunsaturated fatty acids (PuFA) is based on the reduction of the one or increase of the other shall be irrelevant here [8,10,11,12]. Beyond these nutritional concerns, there is further motivation to disembark from triglyceride crystal structured lipid phases. Chemical modification (hydrogenation) can be used to convert unsaturated fatty acids, present in liquid oils, into saturated fatty acids that constitute consequently high melting fats [3]. Since chemically modified food ingredients are less attractive to consumers and they often link hydrogenation to trans-unsaturated fatty acids irrespective of the process execution, this route is currently not promising.

Consequently, the past decades have seen a surge in the use of SaFA originating from tropical oils. That mainly concerns palm oil, which has not only due to food uses developed into the most produced oil on the globe: more than 70 Mio tons per annum. Numerous organizations, to name a few, the Round Table for Sustainable Palm Oil (RSPO), the Malaysian Palm Oil Board (MPOB), Forum Nachhaltiges Palmöl (FONAP), and Non-governmental organisations such as the World Wildlife Fund (WWF), made efforts to improve the sustainability of palm oil. For example, the position of consumers in Europe toward palm oil remains at least controversial [8,13]. The consumer’s position has resulted in an increasing demand for healthy, organic, sustainable, and regional food products. That has led to the desire to offer products comprising alternatives to current structuring routes based on crystalline triglycerides, mainly originating from palm oil or other tropical oils. 

Oleogelation, or more precisely non-triglyceride oil structuring, offers an alternative route to entrap liquid oils in a macroscopically semi-solid lipid structure. Depending on the structuring mechanism, this route could offer independence from tropical oils and the delivery of structured lipid phases with improved nutritional profiles. In essence, the structuring of liquid edible oils can be achieved through different routes: crystalline particles, self-assembled networks, polymer networks, and emulsions or foams. The interest in this field is well documented by the number and diversity of related publications: more than 3000 on the topic of oleo-/organogelation in the past 20 years. Even though many structuring systems have been reported and many patents have been submitted, the number of product applications in food remains surprisingly small. 

Even though this contribution discusses the applicability of oleogels in foods, the aim is not to generate another general review of the area. Next to the reviews of the past years [14,15,16,17,18,19,20,21,22,23,24,25,26,27,28], just the year 2020 has seen more than nine reviews discussing oleogels related to foods [8,29,30,31,32,33,34,35,36]. During this period, also more than 700 original publications relating to oleogels and food have been published. Consequently, this contribution discusses the different technical routes of oleogelation only briefly. In contrast, the necessary non-technical conditions for a successful oil structuring system will be discussed. The emphasis of this contribution is the characterization of the gels. The methods utilized in many contributions are reviewed and discussed concerning their relevance for product applications. For some characteristics, alternative approaches are suggested. Hence, the manuscript is aiming to at least stimulate discussion on the methods typically applied. Additionally, this contribution should give some guidance for future characterization to allow for better comparability and compatibility of the data generated by different labs. A more standardized characterization relevant for applications will undoubtedly benefit the process of bringing oleogels towards implementation. 

## 2. Non-Triglyceride Structuring Routes

The field concerned here is often referred to as organo- or oleogelation. The application of these terms creates a problem in terminology. The lipid phases fulfilling specific requirements in food applications that cannot be meet by liquid oils are not necessarily gelled. Anyhow, the question of whether highly viscous systems should, despite the absence of a linear visco-elastic region, be considered oleogels is of minor importance. In the first place, structured lipid phases are concerned that immobilize liquid oil and can be described as soft solids macroscopically. This feature is essentially based on the presence of at least two distinct phases. Typically, a dispersed phase is aggregating so that a three-dimensional scaffolding is established, which, analogous to a sponge, immobilizes liquid oil. The nature of the aggregating material can be manifold, such as crystalline, amorphous, self-assembled, or liquid crystalline. Next to the oleogels based on a fragile structure dispersed in the continuous oil phase, oil-in-water emulsions are considered oleogels, since these potentially deliver the functionality of structured lipid phases. These emulsions with preferentially high internal phase volume (HIPE) do not concern structured oil but can be seen as mayonnaise analogs instead, rendering the oil phase discontinuous by compartmentalization. Due to their utterly different nature, these oleogels will not be further considered in this contribution.

The different routes to structure the lipid phases have been discussed in depth elsewhere [8,14,15,16,17,20,27,28,29,30,31,32,33,34,35,36]. There are several attempts to categorize the different structuring systems. Essentially there is a distinction between oil gelators being either low molecular weight (LMWOG) or polymeric. A third category covers emulsions and foams. The category of LMWOG includes crystalline and fibrous network structures. In both categories, mixed or single component systems are often distinguished. Unfortunately, these terms remain ambiguous in this context. The term “single component” suggests an ingredient with a very high concentration of a single molecule. Regarding this assumption, the interpretation of thermodynamical information is defined by fundamental relations. Most often, “single component” refers to a class of molecules, if anything. For example, that renders the term melting point inappropriate for any natural wax because it is a multi-component mixture. Consequently, these terms should be used cautiously. 

Next to the fibrous or crystalline material, the microscopical sponge-like structure can be based on polymeric networks. Here, typically, a distinction between direct and indirect dispersion is made. In this case, the use of the terms “pure” and “mixed” should be considered carefully. Table 1 illustrates this categorization and gives some examples (adapted from [14,15,16,17,20,27,28,29,30,31,32,33,34,35,36]).

When utilizing crystalline material, it is fair to assume that the gelation mechanism is similar to the reference, fat crystals of high melting triglycerides. The effectiveness of structuring oil varies significantly for crystals of different TAG compositions. That variability originates in, among others, crystal size, crystal habit, and crystal–crystal interaction. The similarity to the TAG-based approach is apparent when crystals are generated from materials containing saturated aliphatic chains. For example, these molecules are monoglycerides, diglycerides and their mixtures [37,38,39,40], fatty acids, fatty alcohols and their mixtures [41], sorbitan esters [42], wax esters [43], and waxes [44]. That implies that at any given temperature, a distinct solubility of the structurant in the liquid phase is given [45]. In addition to the structuring efficiency—hardness increment per fraction dispersed phase—the solubility directly relates to the efficiency of the structuring agent’s dosage. Low solubilities at ambient temperature correspond to the high melting points of pure components in a first approximation. However, with most structurants being mixtures, this relation is less clear. Unfortunately, the high melting temperature or better dissolution temperatures implicitly carry the risk of less desirable organoleptic properties of the structured lipid phase due to the formation of waxy films. In gels based on at least two different classes of molecules, the basic structuring mechanism is less obvious. In most cases, it is not identified how far the synergistic effects found for the interacting components are based on co-crystallization, sequential crystallization generating sintering, or simply crystal habit modification. Examples for these systems are specific combinations of fatty acids and fatty alcohols [41], mixtures of waxes and lecithins [46], sorbitan esters and lecithins [23,47], and combination sterols and monoglycerides [48]. For these systems, the individual components’ solubility in the oil phase has to be considered carefully because the relative solubilities might vary on temperature change. Waxes, which were mentioned in the previous category as a single component, should be considered here because they contain a significant fraction of different species, such as wax esters, fatty acids, and fatty alcohols [49]. It should be mentioned briefly that for particle gels, the structural elements are usually subject to crystallization processes. These are generally well understood and thus influence the structural elements by variation of cooling rate and shear. That can be used either deliberately to establish the gel itself or be considered during the food product manufacturing process. 

Alternatively, self-assembled fibrils have been found to structure liquid oil. In this case, the fibrillar structures have to assemble such that a 3-dimensional network evolves [50,51,52,53]. On the one hand, it can include fibrils of single components such as 12-hydroxystearic acid (HSA) [54,55] or ricinelaidic acid [56]. On the other hand, binary fibrils such as those based on γ-oryzanol and β-sitosterol [52,53,57] or those based on tocopherol and lecithin [50] have been discovered. Lastly, the structuring of oil can also be achieved by liquid crystalline elements, as exemplified by the combination of lecithin/water or monoglyceride/water. 

Next to the particle gels based on crystals, polymers such as ethylcellulose, which are dissolvable in oil, reportedly generate oleogels [58,59,60,61]. It is much less clear for these systems than for crystal-based gels how the establishment of the structure can be influenced and possibly controlled by processing. Systems based on structurants that are practically not soluble in oil represent a very different category of structured lipid phases. In these cases, the three-dimensional scaffolding or building blocks that host the liquid oil are mostly fabricated in the absence of the oil phase. Examples of this approach are protein-based oleogels [62,63] and oil structuring based on κ-carrageenan [64]. For these types of oleogels, the structure is typically fixed in a water-continuous system. Subsequently, the continuous water phase is substituted with the oil phase. This substitution has been demonstrated successfully by applying various techniques based on either solvent exchange or an intermediate aerogel production through drying [62,64]. 

The examples given illustrate that there are several routes to structure liquid oil. However, from the brief discussion, it is apparent that the identification of successful substitutes for high melting triglycerides is anything but trivial. The requirements for structurants used in industrial food applications have been formulated previously by Co and Marangoni [26] and Bot and Flöter [19,65]. Materials considered for oil structuring purposes should preferentially:

- Be food grade
- Provide a functional structure to edible oil
- Be affordable so as not to excessively increase the price per volume lipid phase
- Not interfere with other ingredients in a product
- Be versatile, having the flexibility to manipulate product properties such as disintegration temperature
- Ensure similar taste and mouthfeel to the reference product.

This list of criteria is a formulated multidimensional ambition rather than a tick-off list since it is practically impossible to meet all criteria. Anyhow, the list is missing another crucial parameter for any successful product introduction: consumer acceptance. Consumer acceptance remains crucial and cannot be achieved if the substitute does not deliver a similar sensory sensation or other exceptional benefits. Considering that most oleogel systems have an entirely different disintegration characteristic than traditionally structured lipid phases, matching organoleptic perception is challenging. Additionally, the substitution of TAG-structured lipid phases driven by consumer concerns regarding chemical processes, sustainability, and regionality should satisfy consumers’ expectations concerning these aspects. 

The vast body of research touched upon is practically insurmountable. Despite the large number of contributions to the field, the comprehensive knowledge generated remains fragmented. Results from different labs and on different structuring systems do not give much guidance for food developers interested in applying oleogels. The first step toward an improved comparability of results would be a more standardized characterization of oleogels. In doing so, it would be desirable to verify the relevance of oleogel characteristics determined for product applications. 

## 3. Characterization of Oleogels

The characterization of structured lipid phases could aim for either an improved understanding of the oleogel itself or the determination of relevant properties for product applications. Since the field of non-triglyceride structuring in foods can still be considered in its infancy, no substantial application knowledge in food products exists. That leaves the question of how far a particular characteristic is relevant for application open for debate. Still, a specific set of characteristic properties that distinguish different structured liquid phases from each other and appear meaningful has emerged. These can consequently be found in most publications concerning oleogelation. Even though not well defined, the critical concentration indicates the lowest concentration of the structurant yielding a non-leaking, non-flowing soft solid. Secondly, a measure for the structure’s ability to hold liquid oil is expressed as oil-binding capacity (OBC). It is mostly expressed as the fraction of oil remaining in the structure after applying external forces, mostly centrifugal force. Several ways to characterize the structure are considered, most often macroscopic hardness and rheological behavior.

Additionally, most contributions include the transition temperatures from liquid to gel (sol–gel) and gel to liquid (gel–sol). The observations reported often cover attempts to elucidate the underlying microstructure of the gels through visualization. That mostly covers light microscopy, which is often complemented by electron microscopy. In some cases, the structure elucidation attempts utilize more advanced methods such as X-ray diffraction, Fourier-transform infrared spectroscopy (FTIR), and alike. Even though these properties’ determination should allow deriving a comprehensive understanding of the different systems, this is rarely the case. To illustrate that, the experimental strategies used most often are discussed in the context of the information sought. 

### 3.1. Critical Concentration—Solubility and Crystallization

**Figure 1:**
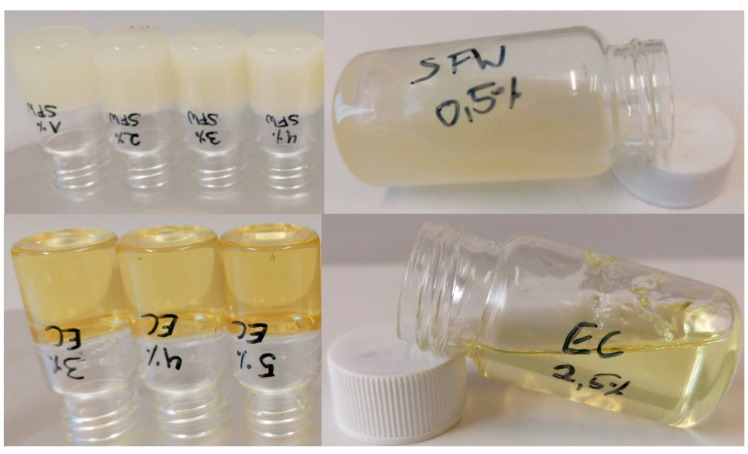
Upside-down test to determine critical structuring composition. After stabilization (48 h, 5 °C), sample vials with different compositions (1 to 10% (*w*/*w*) in 1% steps) are turned upside down. Stable composition left, instable composition at intermediate composition right. Top row sunflower wax in canola oil (0.5%); bottom ethylcellulose (100) in canola oil (2.5%) [66].

The critical concentration is typically defined as the minimum concentration of a structurant in oil that results in a non-liquid state. This postulation is of rather arbitrary nature. This becomes obvious when the different experimental protocols applied are considered. In simple terms, the most applied method involves the following steps. Different concentrations of the structurant are dissolved in the oil phase at sufficiently high temperatures for the respective concentration to generate a solution. Often, a concentration series with steps of 1% is used. The solution is subsequently cooled down, mostly uncontrolled under ambient conditions, for elongated periods of time, between 24 h and several days, in order to allow for the possible development of a gel. However, sometimes, lower temperatures and longer periods of time are applied. Once this period is elapsed, the vials containing the samples are turned upside down. The critical composition is determined by visual inspection. It is the lowest concentration resulting in a sample that does not flow down in the vial. For a typical indication, see Figure 1. Often, the lowest concentration yielding a gel is taken forward for further characterization of the structuring system.

This method is practical, but it deserves some attention concerning what is determined. Considering particle gel, the processes involved to deliver a non-flowing gel essentially include the generation of supersaturation to induce nucleation and crystal growth followed by aggregation of the crystals to form a space-filling, oil-binding network. The supersaturation exerted is directly related to the solubility at the temperature of storage. To what extent solid equilibrium levels are reached results from the interplay of supersaturation, crystallization kinetics, and stabilization time. Next to the volume fraction of solid material, its spatial distribution and crystal sizes play a vital role in this test’s performance. In essence, this determination of the critical composition monitors a sol–gel transition depending on kinetics and is hence not fully suited as a characteristic property. 

**Figure 2:**
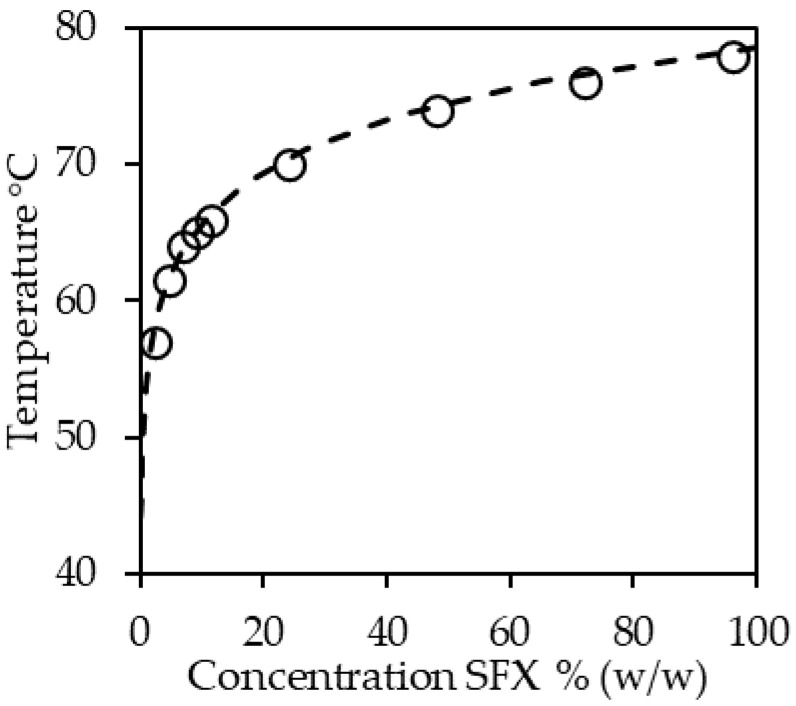
Dissolution temperature of sunflower wax ester (SFX) in canola oil. Temperatures determined by differential scanning calorimetry (DSC). Temperatures exclusively assigned to waxesters present. Dashed dotted line calculated solubility curve for the waxester fraction in the sample assuming ideal solubility, adapted from [67].

In contrast to the sol–gel transition, the determination of solubilities generates detailed reproducible data. In this case, it needs to be safeguarded that equilibrium or at least long-term metastable states are studied. Determination of the solubility curve over a broader concentration and temperature ranges is not only relevant for the determination of the amount of structurant, which will be dissolved in the liquid phase at any temperature under equilibrium conditions. Assuming that the structurant is a pure component, which in most cases is not the case, the solubility curve straightforwardly allows determining the amount of solid material in a sample at any given temperature by simple mass balances. A benefit of this approach is the possibility to formulate the relation between fraction of dispersed phase and temperature, which is a common characteristic used in fat technology. The fact that structuring systems are often mixtures compromises this approach based on simple solubility calculations. However, in some cases, mixed structurant systems can be treated as pseudo-pure components or reduced to its main component [67]. In essence, the solubility curve is the accumulation of the gel–sol transition temperatures. As illustrated in the figure above (Figure 2), systematic gathering of these data allows for inter- and possibly extrapolation.

**Figure 3:**
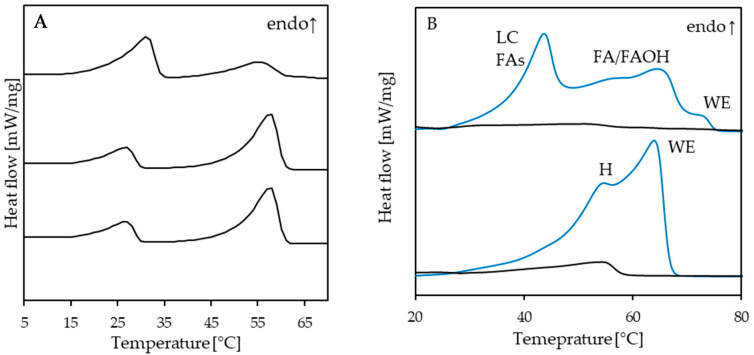
DSC heating thermograms; stabilization for 48 h at 5 °C, scan rate 5 K/min. (**A**): ternary mixtures composed of 90% (*w*/*w*) MCT oil, and 10% of mixtures of pure (>99%) wax esters; from top to bottom 2:1; 1:1, and 1:2 of palmityl–myristate (C16 FaOH and C14 FA) and behenyl–arachidate (C22 FaOH and C20 FA). [69]. (**B**): Blue curves: pure bees wax (top) and bees wax hydrolysate (bottom), black curves: their corresponding 12% *w*/*w* oleogels in canola oil. WE—wax esters, H—hydrocarbons, LC FAs—long-chain fatty acids, FAOH—fatty alcohol [67].

The knowledge of the solubility curve has another benefit considering process design. The data shown in Figure 2 illustrate the problems emerging from the fact that structurants are mixtures. The temperatures displayed are derived from a thorough analysis of differential scanning calorimetry (DSC) heating curve data of different sunflower wax solutions that were crystallized and stabilized. Peaks were assigned to the wax esters present in sunflower wax, and consequently, the wax ester concentration and not the wax concentration is considered an x-value. For the modeling, the wax esters present in sunflower oil, which display a distribution in constituting fatty acids and fatty alcohols, were treated as a pseudo-pure component. The curve shown is calculated based on the enthalpy of fusion and melting point temperature optimized to the experimental values and the equation for ideal solubility [67]. However, this approach falls short of adequately describing the complex solid–liquid phase behavior of sunflower wax and oil in detail. Anyhow, the methodology is reproducible and allows estimating supersaturations. These data are beneficial in comparing different experimental data and could give guidance to process design. Methods to determine the solubility of a high melting structurant in oil are abundant. Among others, optical methods such as turbidity [68], rheological [67,69], or thermal methods can be used to determine both the sol–gel and gel–sol transitions. In any case, it is crucial to ensure adequate sample stabilization when equilibrium solubilities are to be determined. The fact that equilibrium states are process-independent offers the possibility of evaluating stabilization procedures [6]. The determination of dissolution temperatures is often performed by differential scanning calorimetry (DSC). To discuss the protocol in detail here is beyond the scope of this contribution. Anyhow, the sample stabilization and scan rates have to be chosen adequately for the sample at hand to deliver reliable results. An additional benefit of an elaborate DSC study is the elucidation of the more detailed crystallization behavior of the structurant. That is less enlightening when dealing with a pure structurant but highly relevant for hybrid systems, possibly indicating either a single mixed solid phase or multiple co-existing solid phases. Figure 3A shows the melting thermograms of mixtures of medium-chain-triglyceride oil (MCT) and the wax esters palmityl myristate (C16 FaOH and C14 FA) and behenyl arachidate (C22 FaOH and C20 FA).

All samples contain 90% (*w*/*w*) oil and 10% wax esters (*w*/*w*) with varying ratios (from top to bottom 2:1; 1:1, and 1:2). The data indicate that the two structurants crystallize separately, which is also confirmed by analyzing the evolution of the enthalpies involved [69]. Figure 3B shows thermograms of more complex mixtures. The black curves also characterize samples with 10% structurant concentration in canola oil. The blue curves are the heating curves of the two structurants, beeswax, and beeswax hydrolysate. These multi-component structurants contain the same composition with respect to the presence of fatty acids and fatty alcohol moieties. Figure 3B illustrates that it becomes increasingly difficult to assign specific peaks to molecular species present in the mixture. However, as pointed out by Wettlaufer et al. [67], these efforts are considered worthwhile because the deeper insight into the contribution of individual molecular classes can create a more detailed understanding. 

However, the data allow for determining the sol–gel transition temperature curves similar to Figure 2. As illustrated by Figure 2 and Figure 3B, the detailed analysis of the thermograms necessitates in-depth knowledge of the composition of the structurants. In particular, for wax-based oleogels, the understanding of the structuring will remain phenomenological as long as waxes are not specified in more detail. Currently, the elucidation of the relation between wax composition and functionality is insufficiently pursued. Irrespective of their very different and complex composition—at least mixtures of wax esters, fatty acids, fatty alcohols—waxes are predominantly treated as pure components and largely remain unspecified, despite laudable exceptions [49] and the availability of typical compositions [70]. 

For systems that do not involve crystallization but the self-assembly of binary structures, it is undoubtedly also recommendable to study solubility. However, that might not be possible by DSC due to the small energies relating to the phase transitions. Indeed, the dissolution process underlying the gel–sol transition, which is a continuous process on temperature increase, complicates matters. In these cases, other methods are more suitable [71]. Additionally, extrapolations to zero-dispersed phase fraction values based on measurements of a two-phase (solid or dispersed phase and liquid) system along concentration series could be useful. The rheological characterization of samples is often used to study the crystallization behavior on cooling [71,72], but it is rarely reported as a scan of increasing temperature to study the gel–sol transition [69].

Nonetheless, the authors believe that understanding the solubility of the structuring system’s individual constituting components is a prerequisite for the sound interpretation of observations made in hybrid systems. Useful solubility data would enable determining the fraction of the dispersed phase at any temperature. Assuming a simple crystallization behavior of a single component, this can even be done by textbook knowledge. Currently, this property is sometimes wrongly assessed in non-TAG oleogels applying p-NMR methods established exclusively to determine the solid fat content (SFC, AOCS Official Method Cd 16b-93). Since the level of solid material is a meaningful property, it has to be pointed out that the application of methods designed for solid triglyceride structures can only deliver quantitative data if an adequate calibration has been performed.

### 3.2. Gelation, Solution-to-Gel Transition

**Figure 4:**
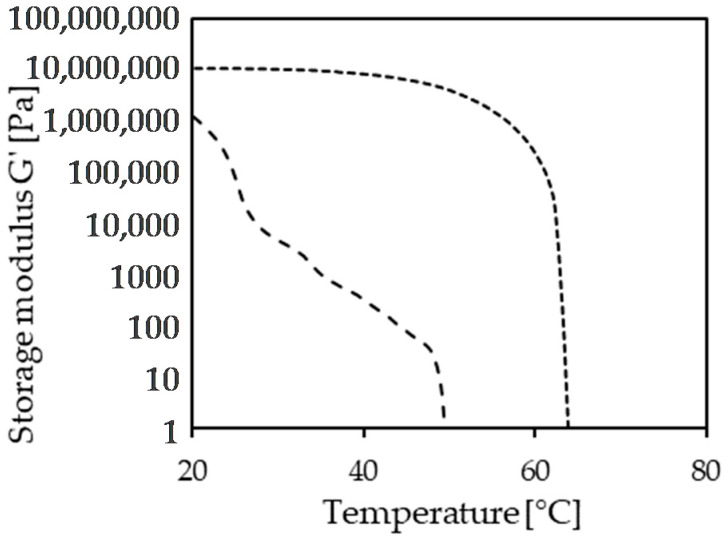
Storage modulus on cooling temperature sweep, 12% structurant in canola oil, short dashed: bees wax hydrolyzate, long dashed sunflower wax. Cooling at 5 K/min at constant strain 0.1% and angular frequency of 10 rad/s (plate/plate geometry, MCR 302, Anton Paar) [67].

Depending on the type of structuring system—from crystals to binary self-assembly—the structure generation’s kinetics can vary greatly. This property could have major implications for the possible application in food products. A summary of the role of fat phases in food products is given elsewhere [8,73]. In some cases of product manufacturing a semi-solid lipid material is needed as the starting material. Here, the kinetics of the developing structure is of little importance, as documented by Zetzl for the application of oleogels in frankfurters [74]. Similarly, the gel development kinetics is less critical in ice cream due to the low-temperature storage during the ripening step [75,76]. That might hold as well for the application in baked goods, where the lipid phase effects are primarily seen in the dough and hence before baking. In other products such as confectioneries and spreads, the lipid phase must change its structure during the manufacturing process from liquid to semi-solid [73], and kinetics could hence be critical. To characterize the sol–gel transition in a relevant way remains a major obstacle because the shear rate, temperature profile, and the product matrix influence this process. These conditions vary significantly for different food manufacturing processes. Unfortunately, numerous contributions omit this area entirely. Even though it is impossible to reproduce specific application scenarios, the combination of gel–sol transition data (solubility curve) and sol–gel data is vital for developing application strategies for alternative structured lipid phases. To this end, both DSC and rheology offer straightforward characterization methods. The application of DSC suffers on one hand from the lack of shear during cooling, rendering crystallization temperatures sometimes unrealistically low. On the other hand, systems not involving crystallization in the structuring process might not yield sufficiently strong thermal signals. Consequently, the most reliable practical way to characterize the sol–gel transition is by rheology [44,49,67]. The experimental execution offers choosing specific cooling rates and modes of shear force application (rotation, oscillation). Hence, it is recommended to vary these parameters to derive a system-specific procedure that delivers the most robust results. Figure 4 shows the storage moduli of a sample containing 12% (*w*/*w*) of either beeswax hydrolyzate or sunflower wax in canola oil. Liquid samples were cooled at 5 K/min at constant strain [67]. 

Beeswax hydrolyzate (short dashed) shows a stepwise crystallization since it is a complex mixture containing fatty acids, fatty alcohols, and wax esters, all of varying carbon chain length. In contrast, the sunflower wax data (>95% wax esters, long-dashed line) indicate the co-crystallization to a single solid phase. This observation is in line with the data shown in Figure 2. 

### 3.3. Macroscopic Properties: Hardness and Deformation

The macroscopic properties of the soft-solid, possibly most important for product developers, can be characterized in many ways. Predominantly, the hardness of oleogels is assessed by penetration measurements. Typically, the force necessary to have a cylindrical body penetrate the gel is monitored. Depending on the gel and the details of the experimental setup, other characteristics such as cohesiveness and brittleness can be determined as well. Unfortunately, these characteristic data are often gathered so that no dose–response relation can be formulated. 

**Figure 5:**
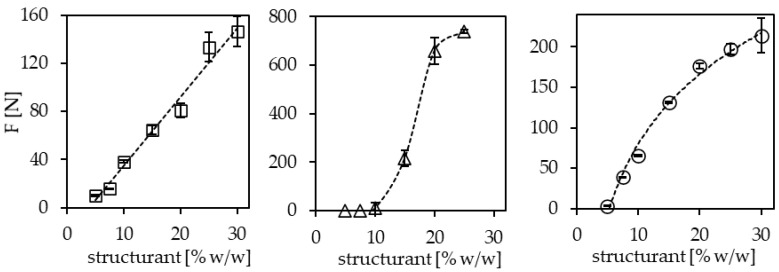
Hardness determined by penetration test as a function of structurant concentration in canola oil: squares: sunflower wax; triangles: 1:1 molar mixture sitosterol/oryzanol; circles: ethylcellulose 100 cP. Samples stored for 7 days at 5 °C; stabilized for 2 h at 22 °C before analysis.

Even though the hardness is a function of the process applied during gel formation, it is advocated here to evaluate the hardness contribution in a more comparable way. At present, the structuring potential of a system is often assessed as hardness at the critical composition, which is often indiscriminately determined, as pointed out above. Alternatively, hardness measurements on different structurants at a fixed concentration in oil allow comparison of the structure generation per %-structurant dosed but ignore solubility differences. To characterize the potential of structurants more comprehensively, the determination of the incremental hardness contribution appears beneficial. These data could help in product development processes to select compositions to yield a specific hardness. Acknowledging that the concept of incremental hardness contributions builds on the doubtful assumption of a linear relationship [33,34], it certainly yields useful insight into the relative potency of different structuring systems. To derive Δ(hardness)/Δ(disperse phase), it is not necessary to determine how much of the disperse phase is present in the system. This concept is illustrated in Figure 5 and reveals that the linearization of slopes at the lowest concentrations allows the compute structuring efficiency values, Δ(F_max_)/Δ(concentration structurant). 

At 22 °C, these are for the systems studied, 5.7 N/% (*w*/*w*) structurant for SFX, 12.4 N/% (1:1 molar mix sitosterol/oryzanol), and 48.7 N/% for ethylcellulose (100 cP). Another benefit of these data is the possibility to extrapolate the linear approximation to a zero-hardness value, which relates to the critical concentrations at 22 °C. Values determined are 3.8% (*w*/*w*) for SFX, 4.6% for sitosterol/oryzanol, and 9.7% for ethylcellulose. These are in line with the respective critical concentrations given for these structurants elsewhere [59,77,78,79]. 

In addition to hardness, the deformation characteristics of the gels are of interest. These can be assessed using back extrusion experiments or any other deformation test. A more detailed analysis of the structural elements and the breakdown characteristics can be gained by further rheological analysis. The literature offers a wide range of settings to characterize oleogels, covering temperature, frequency, amplitude sweeps, and long-term monitoring [80] to gain insights into the structural nature of the three-dimensional scaffolding. Even though the application of detailed rheological protocols on oleogels and their disintegration processes would be of great value, this is rarely reported. That is because either the stabilization of oleogel samples in the rheometer is impractical because of duration or the risk of generating artifacts with any procedure to transfer the gelled samples onto the rheometer.

### 3.4. Microstructure

Considering the range of systems studied in the field of structured lipid phases, the elucidation of the structure and identification of the scaffolding’s building blocks and their mutual interactions is a humongous task. Only a few systems have been studied in such detail so that consistent hypotheses on their microstructure have emerged. These are gels based on the binary system of β-sitosterol and γ-oryzanol [72,81,82,83] and those based on ethylcellulose [58,60,84,85]. For the β-sitosterol and γ-oryzanol system, the structural hypothesis is a three-dimensional network of self-assembled micro-fibrillar tubules, 8 to 10 nm in diameter depending on the definition of the tube outer diameter. Interpretations of the X-ray diffraction patterns, neutron scattering data, atomic force microscopy, electron microscopy, and even molecular modeling coherently support this hypothesis [57,72,83].

**Figure 6:**
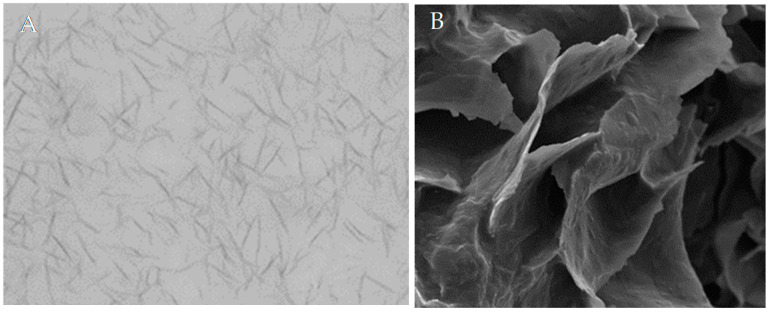
Visualization of the microstructure of 10% sunflower wax in canola oil. (**A**) Polarized light microscopy, image height 500 µm. (**B**) Cryo-SEM image height 125 µm. The agreement of the structural hypothesis derived is not intrinsic. For details of the experimental method, see [66].

Similarly, the structural hypothesis given by Zetzl [58] for ethyl cellulose-based gels has been reconfirmed repeatedly using different experimental techniques. However, the deduction of a structural hypothesis is often attempted based on insufficient data. In particular, in interpreting polarized light microscope images, one should be cautious not to overinterpret large-scale agglomerates due to the risk that the limited resolution does not allow the identification of objects, such as the above-mentioned tubules. Since all methods applied have an individual risk of artifacts due to, for example, preparation procedures, it is most important not to rely on a single method. It has to be stressed here that the consistent interpretation of the thermo-physical data (crystallization and rheology) and structural hypothesis based on visualization in the broader sense is far from trivial. This is illustrated in Figure 6. Both images depict an oleogel of 10% (*w*/*w*) sunflower wax in canola oil [67]. 

The left LM-micrograph image is typically interpreted as the arrangement of needle-like crystals, while the electron microscope image on the right indicates the formation and sintering of platelets.

### 3.5. Gel Integrity-Immobilizing Liquid Oil

In the research related to non-triglyceride structured lipid phases, the so-called oil-binding capacity (OBC) is considered an essential property of oleogels. Before discussing the different methods to evaluate OBC, it is worthwhile to review this property’s underlying concept. To this end, the functionality of a structured lipid phase in a product shall be considered. First, we assume that a structured oil phase is applied in oil-in-water emulsions. In that case, the function is supposedly either in the suppression of emulsion break-up [76] or limitation of coalescence and resulting droplet size increase, e.g., in ice cream. Additionally, in these systems, the structured dispersed phase influences, to a limited extent though, the macroscopic properties shown for yogurt [86]. For products with continuous lipid phases, the non-triglyceride structure has to supply the same “sponge-like” immobilization of the liquid oil as fat crystal networks do for example in spreads or chocolate. In other products, e.g., baking applications, the incorporation of lipid phases into the dough involves shearing and dispersion. Consequently, the gel domains have interphases with aqueous slurries and other matrix elements. That leaves the question of what type of integrity of the structured lipid phase is needed. In this light, a compression test could also be considered an OBC test. One approach could be the compression of the scaffolding so that the oil is exuded from the volume-reduced three-dimensional network. 

In such a hypothetical test, the question arises of which contributions are relevant for the exudation. Here, a few simple answers spring to mind: (1) the exclusive compression of the scaffolding, directly relating to the very scaffolding constituting an aerogel; (2) the resistance to flow through a porous matrix; (3) surface tension forces due to the concave liquid surface concerning the gels’ environment; (4) adhesion of the oil to the surface of the scaffolding. Considering compression (1), the dominant factor of the OBC should be assessed as a compression test, rendering testing OBC would be obsolete. However, assessing available data does not confirm this relation. 

The second hypothesis has been studied superficially in our lab, and a change in the viscosity of the continuous phase does not correspond to the changes in the gel’s behavior (unpublished data). The contributions listed under (3) and (4) are more difficult to unravel. In both cases, the surface tension between the scaffolding material and the oil plays a significant role. Considering the gel’s outer surface, the contact angle between the scaffolding surface and oil considering the outer atmosphere are essential in analogy to capillary effects. Preliminary experiments concerning the effect of the interfacial tension between gel and surrounding atmosphere have not revealed significant gel integrity effects yet (unpublished data). Compression experiments were carried out with the gel specimen submerged in air (a common practice), water, and a mixture of water and emulsifiers to modify the interfacial tension. These studies did not reveal any significant differences (unpublished data). The adhesion of oil to the gel sample’s inner surface (4) is more challenging to examine. Anyhow, this could be expressed as mobility of the oil within the matrix. Even though one could be tempted to study this at a microscopic level, the rate of perfusion possibly offers a meaningful assessment at a macroscopic level. 

Taking the above into account, the common practice to assess OBC by centrifugation is questionable. The method is typically executed such that samples are centrifuged at high centrifugal forces (>3000 g) for significant periods of time (>10 min). The resulting visually clear supernatant is decanted, and the ratio of the remaining sample volume and the original is taken as OBC. Obviously, OBC determined according to this method is a function of the density difference between scaffolding and liquid oil. It defines the compression force exerted on the scaffolding. Moreover, the method is susceptible to the stiffness of the scaffolding. 

**Figure 7:**
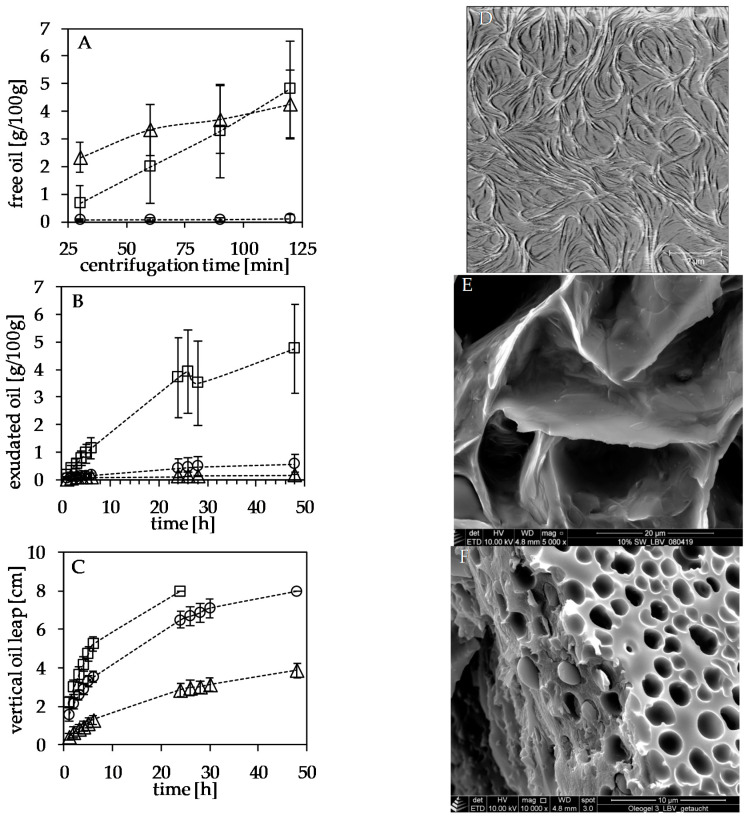
Left: Comparison of different methods to determine oil-binding capacity (OBC): oil exuded as function of time for 10% (*w*/*w*) structurant in canola oil oleogels. (**A**) Conventional centrifugation test (time in minutes), (**B**) filter-paper method block on paper (time in hours), (**C**) filter-paper climb method (time in hours) [66]. Symbols: squares: ethylcellulose, triangles: 1:1 sitosterol/oryzanol, circles: SFX. For details of the experimental procedure, see [66]. Right micrographs of oleogels: (**D**) atomic force microscopy sitosterol/oryzanol (scale bar 2 µm), (**E**) cryo-SEM image of SFX (scale bar 20 µm), (**F**) cryo-SEM image of ethylcellulose (scale bar 10 µm).

Our lab’s earlier work shows that excessive centrifugation did not yield substantial oil exudation in instable products such as liquid margarine (2% high melting fat, 20% dispersed water phase), even though this product suffers from free oil in the eye of the consumer (unpublished data, private communication L. Alderliesten/Unilever). That is an indication for the lack of generating relevant discriminative OBC data by centrifugation. The results reported on palm oil shortenings [87] support this hypothesis. In this work, the product was centrifuged for 4 h at 4000 rpm at 25, 40, and 70 °C. Compared to the enormous differences in temperature, the differences in OBC are minute, ranging from 1.1 to 10.6%. Another approach to quantify the oil-binding capacity of structured fats was reported by Omonov [88]. The analysis is based on a filter paper’s weight increase when a defined block of structured fat is placed on it. In samples containing 10% fully hydrogenated canola or fully hydrogenated sunflower oil in canola oil, very systematic data could be obtained, and the OBC of both samples is distinguishable. Moreover, the effect of variation of the crystallization temperature, inducing different microstructures, is effectively documented in the OBC data [88]. While the centrifugation test is prominently used in the field of oleogelation, variations of the filter paper-based test have also been used in industrial laboratories to evaluate the OBC of spreads [89]. Three structuring systems currently studied in our lab were evaluated head-to-head to evaluate different methods determining the OBC. The centrifugation method and the filter paper method were applied in their typical execution next to modified versions. Modifications were designed taken the considerations outlined above into account. Modifications of the filter paper method were also inspired by the Baumann method [90] which is, for example, used to assess the water uptake of dry starch [91]. The details of the different methods and a comprehensive comparison of the results obtained can be found elsewhere [66]. For example, it was attempted to eliminate the effects of compression of the scaffolding in the centrifugation method by using a vial containing a fine mesh on which the gel sample was placed. Hence, the oil was forced out of the gel and accumulated below the mesh in the tip of the vial. In order to eliminate effects of oil depletion of the gel, topping of the gel with liquid oil was studied as another variation. To allow oil to flow into the gel structure appeared to be beneficial for the discriminative power throughout the different methods applied. In a modification of the filter paper method, the problem of gel adhering to the soaked filter paper during weighing is omitted. Instead of placing the gel sample on a filter paper, a vertical stripe of filter paper is inserted into the gel to generate intense contact. The vertical filter paper is used to monitor the “climb” of the oil. In this design, the gel is also not depleted of oil, because the oil taken up by the filter paper is replenished, since the bottom surface of the gel specimen is submerged into liquid oil. The speed at which the oil rises is monitored carefully. The results obtained using three different methods are depicted in Figure 7. The systems studied all contain 10% (*w*/*w*) of structurant and 90% canola oil. Structurants compared are 1:1 molecular mixtures of β-sitosterol and γ-oryzanol (triagles), ethylcellulose (squares), and sunflower wax (circles). Since the emphasis in this contribution is on the methods used, further details of the systems studied are omitted but can be found elsewhere [66]. Figure 7 also illustrates the substantial differences in the microstructure of the three systems studied. For top to bottom the atomic-force microscopy (AFM) image of the sitosterol/oryzanol gel indicates a network of microtubules, the cryo-SEM image indicates that the sunflower wax results in a three-dimensional arrangement of leafleats, while the cryo-SEM of the ethylcellulose gel reveals a more bi-continuous arrangement. Each data point in Figure 7 is subject to 12 repetitions. The conventional centrifugation method (top) indicates the best oil-binding capacity sunflower wax and the worst for the sitosterol/oryzanol system. Surprisingly, the modified centrifugation method (vial with a mesh) described above indicates another sequence of OBC: sitosterol/oryzanol > sunflower wax > ethylcellulose. The two lower graphs show results on filter paper methods. Both indicate the same sequence of OBC with sitosterol/oryzanol being the most stable and ethylcellulose the least oil binding. What the data further reveal is the improved quality of data obtained by the filter strip method described above. Admittedly, the long experimentation times shown bear the risk that the structurant out of the gel matrix dissolves into the oil reservoir or the filter paper. However, the data in the initial phase (up to 6 h) of the oil climbing up the filter strip indicate a linear relation of height climbed versus time. The slopes computed are 0.18 cm/h for the sitosterol/oryzanol gel, 0.38 cm/h for the sunflower wax gel, and 0.6 cm/h for the ethylcellulose gel.

Taking the data’s systematic behavior, reproducibility, and discriminative power into account, the superiority of the newly proposed filter paper-based procedure is striking and warrants further evaluation methods to determine OBC. Interestingly, the methods based on centrifugation and external suction (block on filter paper) resulted in different sequences of OBC for the structuring systems studied. In summary, it seems fair to advocate that the current methods to study the oil binding capacity deserve a critical review. To incorporate the solubility of the structuring material in the interpretation of data could benefit the interpretation similarly to the discussion on the product structure above. Furthermore, the question of which aspect of OBC as a discriminative gel characteristic is relevant for product applications remains open. 

## 4. Application Tests and Feasibility at Industrial Scale

The successful application of oleogels in lab-scale model products is documented in many papers relating to the area. These efforts are well summarized in the review papers that have been published in the past years [8,14,16,17]. Therefore, it should only be pointed out here that the successful fabrication of a mock-up product in a lab environment is far from being proof of the principle of industrial manufacturability. In this context, a detailed understanding of the structured lipid phase’s functionality during the manufacturing process and in the final product is of utmost importance. That implies understanding at which stage the system has to be structured and if there are kinetic constraints to changes in rheological properties, among other things. The proof that a final product with comparable characteristics to the reference product can be fabricated in the lab does not warrant that the offered technologies are suitable for the industrial production of food products. To this end, as discussed above, characterization methods have to be discriminative and relevant so that they can support a meaningful choice of the routes to pursue future innovations. These methods should give guidance to product developers by supplying the characteristics for a range of compositions and process parameters for different oleogel structuring systems. Additional to the technical criteria discussed above, the future success of non-triglyceride structured lipid phases depends on a few mundane factors. The oil-structuring systems of the future need to be affordable and available in sufficient quantities. Next to these criteria discussed elsewhere [8], consumer acceptance remains a critical success factor for future innovations. 

## 5. Conclusions

The area of oleogelation has seen a period of substantial academic activity. That has resulted in an enormous body of scientific observations and data. However, the uptake of the technologies developed has been meager or at least slow until now, since no product introduction into the foods market has been observed. Reasons for this situation can be manifold. The unavoidable increased cost of raw material for food manufacturers or the limited “true demand” by the consumer slows down innovation. The body of evidence created by the scientific community remains widely inconsistent and possibly not suited yet to enable food manufacturers to act. This contribution has tried to elaborate on the methods applied to characterize structured lipid phases and their relevance for evaluating oil-structuring systems’ performance. Unless the characterization of oleogels is undoubtedly indicating which structuring routes should be followed for specific product applications, it is difficult for food manufacturers to embrace the technologies offered. It was shown that most of the methods applied in the research field are close to the current practice in structured lipid phases. However, the evaluation of different structuring systems should be performed less in a singular way than currently exercised. It is necessary to generate a comprehensive understanding of the structuring systems for product development. That means that flexibility of the composition and dose–response relations need to be identified. The detailed description of the composition of structurants is an essential prerequisite to generate any progress in this area. Next to this, the future establishment of solubility curves and determination of incremental hardness contributions outlined in this manuscript could already mark a substantial step into the direction of comprehensive and comparable data. The newly proposed method to assess the oil-binding capacity addresses how far the characterization methods used are relevant and discriminative enough to deliver meaningful data. This area certainly deserves more attention, since future industrial applications will require methods to warrant functionality through specification. In conclusion, this contribution remains a plea for the critical assessment of alternative routes to oil structuring, taking into account functionality as much as the potential for industrial applicability.

## Figures and Tables

**Table 1:** Categorization of oil structurants. Low molecular weight oleogelators (LMWOG) appear in either crystalline or fibrillar structures. Polymeric oleogels can be distinguished based in the dispersion process.

LMWOG				Polymeric		
Crystalline		Fibrillar		Directly Disp.	Indirectly Dispersed	
Single Component	Mixed System	Single Component	Mixed System		Single Component	Mixed System
monoglycerides		12-hydroxystearic acid		ethylcellulose		protein-
n-alkanes	waxes	lecithins	phytosterol/oryzanol		proteins	polysaccharide
fatty acids	fatty acid/fatty alcohol				MPMC	complexes
wax esters	sorbitan ester/lecithin					
fatty alcohols						

## Data Availability

The data presented in this study are available in article.

## Sample Availability

Not available.
